# Gene coexpression analysis in *Arabidopsis thaliana* based on public microarray data

**DOI:** 10.1016/j.xpro.2022.101208

**Published:** 2022-02-26

**Authors:** Vasileios L. Zogopoulos, Apostolos Malatras, Ioannis Michalopoulos

**Affiliations:** 1 Centre of Systems Biology, Biomedical Research Foundation, Academy of Athens, 11527 Athens, Greece; 2 biobank.cy Center of Excellence in Biobanking and Biomedical Research, University of Cyprus, 2029 Nicosia, Cyprus

**Keywords:** Bioinformatics, Genomics, Model Organisms, Plant sciences, Systems biology

## Abstract

Coexpressed genes tend to participate in related biological processes. Gene coexpression analysis allows the discovery of functional gene partners or the assignment of biological roles to genes of unknown function. In this protocol, we describe the steps necessary to create a gene coexpression tree for *Arabidopsis thaliana*, using publicly available Affymetrix CEL microarray data. Because the computational analysis described here is highly dependent on sample quality, we detail an automatic quality control approach.

For complete details on the use and execution of this protocol, please refer to Zogopoulos et al. (2021).

## Highlights

• Download and quality control of raw microarray data from multiple public repositories
• Normalization of microarray samples using SCAN algorithm and the latest BrainArray CDF
• Creation of a gene coexpression tree using UPGMA hierarchical clustering
• Biological term enrichment analysis in gene coexpression tree subclades

## Graphical abstract

**Figure:**
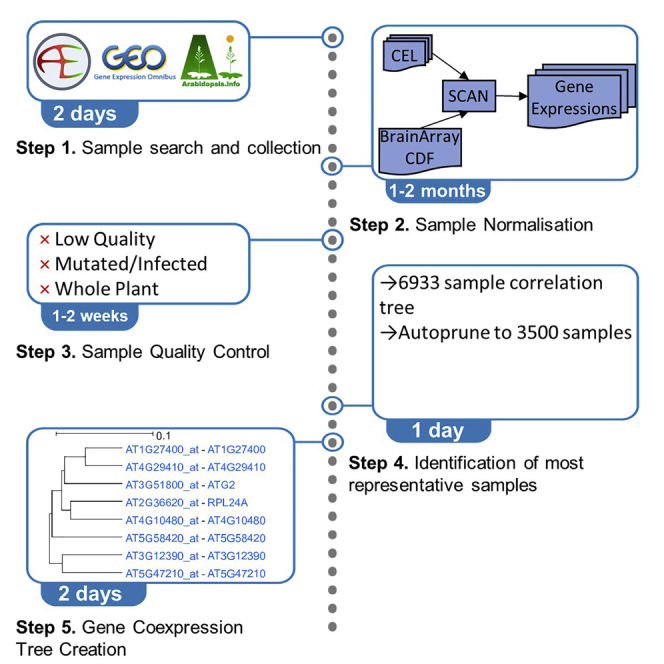

## Before you begin

The protocol below describes the specific steps for performing gene coexpression analysis on *Arabidopsis thaliana* Affymetrix microarray data. The same protocol can be used for any species, provided that at least 20 samples of the same Affymetrix chip are available (Langfelder and Horvath, 2017). By examining the gene coexpression tree created by following this protocol, hypotheses for gene partnership and pathway participation can be made, which can be, in turn, experimentally validated. The advantages described in this protocol include: the hierarchical clustering approach for displaying gene coexpression which outperforms the more commonly used gene coexpression list output (Obayashi et al., 2018; Yim et al., 2013), the automatic quality control procedure and the meticulous representative sample selection which eliminates tissue bias, as well as the usage of modern normalization algorithms along with up-to-date CDFs. Execution of this protocol has resulted in several use-cases, where a gene of interest was discovered to be grouped with genes of similar functions, which are supported by already existing bibliography (Zogopoulos et al., 2021).

### Operating system and necessary hardware

**Timing: 1 h**

Ubuntu 20.04 LTS Linux operating system was installed on a 16-core, 64 GB RAM machine. Ubuntu 20.04 and all necessary software can run on a minimum of 2 GHz dual core CPU, 4 GB RAM and 100 GB hard drive computer setup. However, RAM requirements for the calculation of sample or gene pairwise correlations and hierarchical clustering, depends on the number of available samples and studied genes. At least 64 GB of RAM is recommended for both those steps and that amount should also speed up sample normalization step. The required disk space is proportional to the number of samples and genes. In our case, where 19887 samples and 20430 genes were studied, around 300 GB were required.

1. Perform an installation of Ubuntu 20.04 or equivalent operating system, unless a machine with such an operating system already installed is available.

***Note:*** For software installation on Ubuntu, a sudo user (i.e., a user account granted with root privileges) is required.

After this step, users can proceed either with a Docker installation or by performing a full manual installation.

**CRITICAL:** All commands listed correspond to a Ubuntu 20.04 installation.

### Installation using docker

2. Install Docker by typing the following commands in Ubuntu:
  ***Note:*** This installation is for Ubuntu 20.04. For different operating systems users can refer to: https://www.docker.com/get-started
  a. Install the Docker image of the protocol by typing in Ubuntu:
    sudo docker pull imichalop/act:latest
  b. The container can be run in Ubuntu using:
    sudo docker run -it imichalop/act:latest
  c. Inside the docker container, MySQL needs to be started before starting the analysis:
    service mysql start
  d. Enter MySQL as root:
    mysql -u root -p
  e. default root password is “1234” and can be changed using:
    ALTER USER 'root'@'localhost' IDENTIFIED WITH
    mysql_native_password BY '<password>';
  f. In order to enable local file loading the following command must be typed in MySQL as root:
    SET GLOBAL local_infile=1;
  g. Local Athaliana MySQL database is already created. The local username is “user” with password “1234”. User password can be changed in MySQL as root using:
    ALTER USER 'user'@'localhost' IDENTIFIED BY '<new_password>';
    FLUSH PRIVILEGES;
    ***Note:*** If the user password is changed, then the password field inside /home/ACT/Parsers/config.ini must also be changed
  h. Exit MySQL by typing:
    exit
  i. The system setup is complete and the users can begin the execution of the protocol.

### Full installation

To perform a full installation, the following commands must be typed in Ubuntu terminal:

3. Install system updates, git, unzip and gunzip:

sudo apt-get update

sudo apt-get dist-upgrade

sudo apt-get install git unzip gzip

### Install ACT scripts

**Timing: 1 min**

4. Download the necessary codes for this protocol from GitHub:

git clone https://github.com/imichalop/ACT.git

***Note:*** ACT folder is created automatically through git and all custom programs, scripts and files described in this protocol are included or produced inside.

### Install R & RStudio

**Timing: 15 min**

5. R and optionally RStudio, need to be installed on the Ubuntu machine.
  a. Install latest version of R and necessary dependencies:
    sudo apt install --no-install-recommends software-properties-common
    dirmngr
    wget -qO- https://cloud.r-
    project.org/bin/linux/ubuntu/marutter_pubkey.asc | sudo tee -a
    /etc/apt/trusted.gpg.d/cran_ubuntu_key.asc
    sudo add-apt-repository "deb https://cloud.r-
    project.org/bin/linux/ubuntu $(lsb_release -cs)-cran40/"
    sudo apt-get install r-base gcc make perl libclang-dev libpq5
    libcurl4-openssl-dev libssl-dev libxml2-dev
  b. Install RStudio:
    i. Download the latest version of RStudio for Ubuntu: https://www.rstudio.com/products/rstudio/download
    ii. Install the .deb file using:
      sudo dpkg -i <rstudio-∗-amd64.deb>

### Install MySQL and PHP

**Timing: 10 min**

6. MySQL database management system (DBMS) needs to be installed on the Ubuntu machine, to store and access data, using relevant SQL queries.
  a. Setup MySQL client and server:
    sudo apt-get install mysql-server mysql-client
    i. Access MySQL as root after installation using the following command in Ubuntu terminal:
      sudo mysql -u root -p
      ***Note:*** Press “Enter” when asked for MySQL root password, unless a root password is already set up.
      The following commands must be typed in MySQL environment:
    ii. Set up MySQL root password using:
      ALTER USER 'root'@'localhost' IDENTIFIED WITH mysql_native_password BY
      '<password>';
    iii. Enable local file loading using:
      SET GLOBAL local_infile=1;
    iv. Create a MySQL user account using:
      CREATE USER '<user>'@'localhost' IDENTIFIED BY '<password>';
    v. Create the database using:
      CREATE DATABASE <database name>;
      GRANT ALL PRIVILEGES ON <database name>.∗ TO '<user>'@'localhost';
      ***Note:*** In our example, the <database name> is “Athaliana”.

7. PHP is used in this protocol to run all scripts necessary for data file parsing, format conversion and database access.
  a. Install PHP and MySQL module for PHP:
    sudo apt-get install php-cli php-mysql
  b. Get data from MySQL using PHP:
    ACT/Parsers/config.ini includes the local MySQL credentials and should be changed accordingly.
    username = <user>
    password = "<password>"
    dbname = <database>

### Install MySQL workbench and create the ERD and the tables of the database

**Timing: 30 min**

MySQL Workbench is a visual database design tool to create the Entity-Relation Diagram (ERD) and the tables of the database.

**Figure 1:**
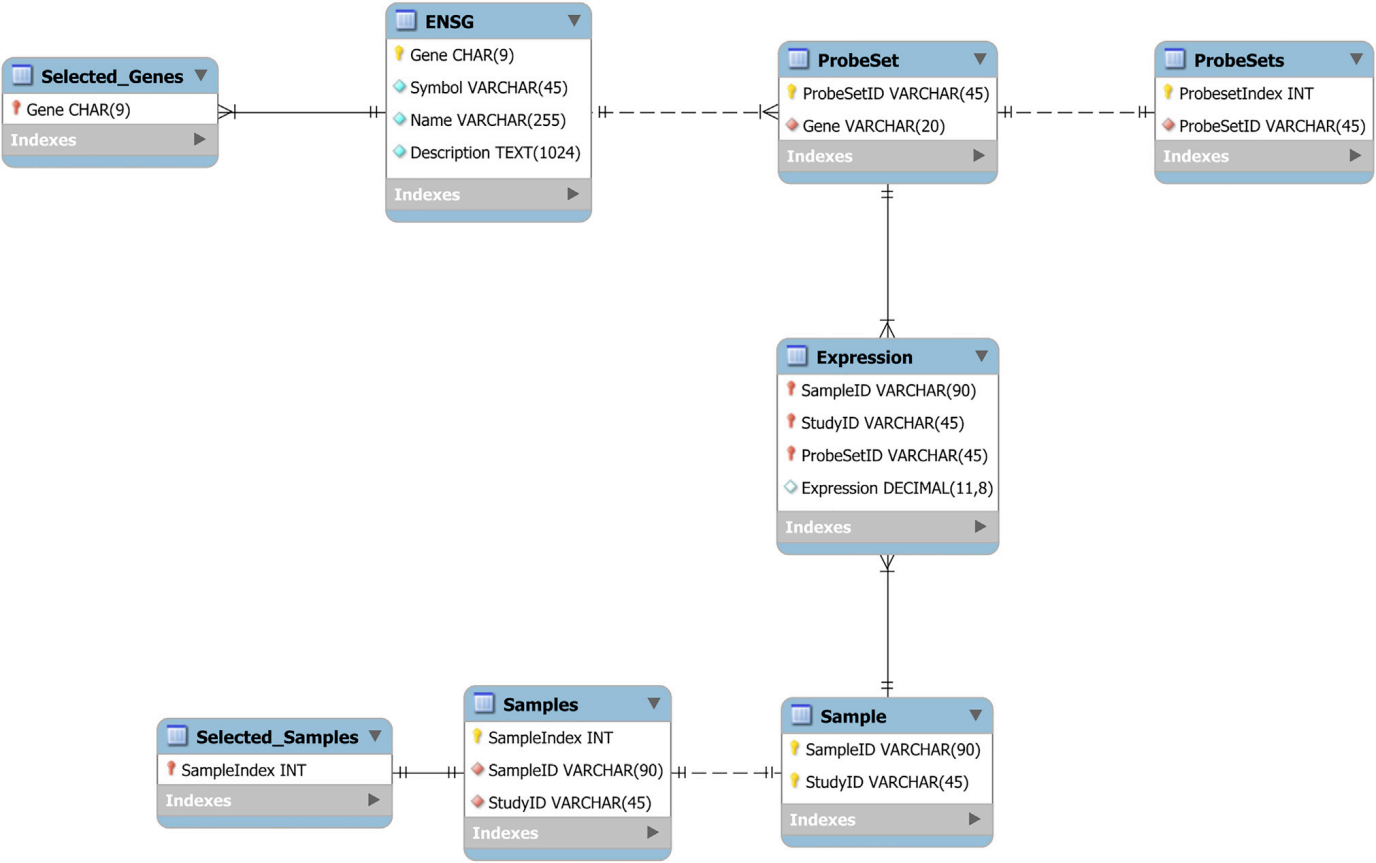
Entity-relation diagram (ERD) in crow’s foot notation of local MySQL database

8. Install MySQL Workbench:
  a. Install necessary packages:
    sudo apt-get install proj-bin libatkmm-1.6-1v5 libcairomm-1.0-1v5 libglibmm-2.4-1v5
    libgtkmm-3.0-1v5 libopengl0 libpangomm-1.4-1v5 libsigc++-2.0-0v5
  b. Download MySQL Workbench for Ubuntu 20.04: https://dev.mysql.com/downloads/workbench/
  c. Install the .deb file using:
    sudo dpkg -i < mysql-workbench-community_∗ubuntu20.04_amd64.deb>

9. The local MySQL database ERD is designed using MySQL Workbench and consists of 8 tables (Figure 1). “Expression” will be used to save the expression values of each probeset in each sample. “Probeset” contains the association between probesets and genes. “Probesets” contains the probesets as well as a unique numeric ID associated with each probeset. “ENSG” contains the AGI code, gene symbol, gene name and brief descriptions of *Arabidopsis thaliana* genes. “Selected_Genes” contains the AGI codes of genes that will be studied. “Sample” contains details of all samples. “Samples” contains the same details as “Sample”, except there is a unique numeric ID associated with each sample. “Selected_Samples” contains the unique numeric ID of the representative samples after quality control is over. Athaliana.mwb is the MySQL workbench file of the proposed ERD in this protocol.
  a. Open a new Ubuntu terminal instance and access local MySQL database from using:
    mysql –u <user> –p <database name> --local-infile
    **CRITICAL:** All commands requiring MySQL will be performed from this distinct terminal instance.
  b. Create the tables by copying and pasting the SQL table creation commands from the MySQL Workbench ERD (Right click an ERD table > Copy SQL to Clipboard).

### Install Array Power Tools

**Timing: 5 min**

10. Install Array Power Tools (Thermo Fisher Scientific, 2020):
  a. Download the APT package for Linux: https://www.thermofisher.com/us/en/home/life-science/microarray-analysis/microarray-analysis-partners-programs/affymetrix-developers-network/affymetrix-power-tools.html
  b. Install Array Power Tools using the following commands in Ubuntu terminal:
    sudo mv apt_∗_linux_64_bit_x86_binaries.zip /usr/local/bin/
    cd /usr/local/bin/
    sudo unzip apt_∗_linux_64_bit_x86_binaries.zip
    sudo rm apt_∗_linux_64_bit_x86_binaries.zip
    cd apt_∗_linux_64_bit_x86_binaries/bin/
    sudo chmod 755 ∗
    sudo chmod 644 axiom_param_conversion.txt apt-annotation-
    converter.config
  c. In Ubuntu terminal, type:
    pico ∼/.bashrc
    to append the following line:
    export
    PATH="/usr/local/bin/apt_∗_linux_64_bit_x86_binaries/bin:$PATH"
    Save and exit the file
    ***Note:*** Replace ∗ in the previous line with the downloaded version of APT.
  d. To add the bin directory to the path, type:
    source ∼/.bashrc
    When you relogin, there is no need to execute this command again.

### Install R packages

**Timing: 1 h**

11. Execute RStudio in Ubuntu terminal as root:

sudo rstudio &

12. Execute the following commands in RStudio environment:
  a. Install latest version of Bioconductor:
    if (!requireNamespace("BiocManager", quietly = TRUE))
     install.packages("BiocManager")
    BiocManager::install()
  b. Install the necessary Bioconductor packages:
    i. SCAN (Piccolo et al., 2012) and oligo (Carvalho and Irizarry, 2010):
      if (!requireNamespace("BiocManager", quietly = TRUE))
       install.packages("BiocManager")
      BiocManager::install("SCAN.UPC")
    ii. InterMineR (Kyritsis et al., 2019):
      if (!requireNamespace("BiocManager", quietly = TRUE))
       install.packages("BiocManager")
      BiocManager::install("InterMineR")
  c. Install Phangorn (Schliep et al., 2017), using:
    install.packages("phangorn")

### Install Newick Utilities

**Timing: 5 min**

13. Install Newick Utilities (Junier and Zdobnov, 2010), typing the following commands in Ubuntu terminal:

sudo apt-get install flex bison

wget

https://web.archive.org/web/20190914014444/http://cegg.unige.ch/pub/newick-utils-1.6-Linux-x86_64-disabled-extra.tar.gz

tar -zxvf newick-utils-1.6-Linux-x86_64-disabled-

extra.tar.gz

cd newick-utils-1.6/src/

sudo cp nw_∗ /usr/local/bin/.

### Install Dendroscope

**Timing: 5 min**

14. Install Dendroscope (Huson and Scornavacca, 2012) using the following:
  a. Visit https://software-ab.informatik.uni-tuebingen.de/download/dendroscope3/welcome.html and download Dendroscope installation script for Linux
  b. Type the following command in Ubuntu terminal:
    chmod 755 Dendroscope_unix_∗sh
  c. Run the installation using:
    sudo ./Dendroscope_unix_∗.sh
  d. During the installation, select the “Check for updates: On every start” option and set max memory usage to at least 16,384 Megabytes.

## Key resources table

**Table:** 

REAGENT or RESOURCE	SOURCE	IDENTIFIER
**Deposited data**		

*Arabidopsis thaliana* Microarray Samples - ArrayExpress	(Kolesnikov et al., 2015)	https://www.ebi.ac.uk/arrayexpress/
*Arabidopsis thaliana* Microarray Samples - GEO	(Barrett et al., 2013)	https://www.ncbi.nlm.nih.gov/geo/
*Arabidopsis thaliana* Microarray Samples - NASCArrays	(Craigon et al., 2004)	http://bar.utoronto.ca/NASCArrays/index.php
Full list of Microarray Samples used	(Zogopoulos et al., 2021)	https://data.mendeley.com/datasets/hgvk669v89/

**Software and algorithms**		

MySQL Workbench	(Oracle, 2021)	https://www.mysql.com/products/workbench/
Single Channel Array Normalization (SCAN)	(Piccolo et al., 2012)	https://www.bioconductor.org/packages/release/bioc/html/SCAN.UPC.html
Brainarray Custom CDF	(Dai et al., 2005)	http://brainarray.mbni.med.umich.edu/Brainarray/Database/CustomCDF/genomic_curated_CDF.asp
Array Power Tools	(Thermo Fisher Scientific, 2020)	https://www.thermofisher.com/gr/en/home/life-science/microarray-analysis/microarray-analysis-partners-programs/affymetrix-developers-network/affymetrix-power-tools.html
InterMineR	(Kyritsis et al., 2019)	https://www.bioconductor.org/packages/release/bioc/html/InterMineR.html
simpleaffy	(Miller, 2018)	https://web.archive.org/web/20201024030658/http://www.bioconductor.org/packages/release/bioc/html/simpleaffy.html
affyQCReport	(Parman et al., 2021)	https://bioconductor.org/packages/release/bioc/html/affyQCReport.html
affyPLM	(Bolstad et al., 2005; Brettschneider et al., 2008)	https://bioconductor.org/packages/release/bioc/html/affyPLM.html
oligo	(Carvalho and Irizarry, 2010)	https://www.bioconductor.org/packages/release/bioc/html/oligo.html
Phangorn	(Schliep et al., 2017)	https://cran.r-project.org/web/packages/phangorn/index.html
Newick Utilities	(Junier and Zdobnov, 2010)	https://github.com/tjunier/newick_utils
Dendroscope	(Huson and Scornavacca, 2012)	https://uni-tuebingen.de/fakultaeten/mathematisch-naturwissenschaftliche-fakultaet/fachbereiche/informatik/lehrstuehle/algorithms-in-bioinformatics/software/dendroscope/
Thalemine	(Krishnakumar et al., 2015)	https://bar.utoronto.ca/thalemine/begin.do
String	(Szklarczyk et al., 2021)	https://string-db.org/
WebGestalt	(Liao et al., 2019)	http://www.webgestalt.org/

**Other**		

Intel(R) Core(TM) i7-8700K CPU @ 3.7 GHz (6 cores × 2 threads)	Intel Corporation	SR3QR
4 TB WD Purple Surveillance Hard Drive	Western Digital	WD40PURZ
2 × VENGEANCE® LPX 32 GB (2 × 16 GB) DDR4 DRAM 2400MHz C14 Memory Kits	Corsair	CMK32GX4M2A2400C14

## Step-by-step method details

### Sample search and collection

**Timing: 2 days**

The main aim of this protocol is to perform a condition-independent coexpression analysis for the species of interest. As such, the microarray samples need to be of various distinct healthy tissues and originate from the same Affymetrix microarray chip.

1. Select an Affymetrix microarray chip for the species of interest. To identify the most popular Affymetrix chip for the species of interest (in our case, *Arabidopsis thaliana* model plant organism), we performed a search in GEO (Barrett et al., 2013):
  a. Visit GEO website: https://www.ncbi.nlm.nih.gov/geo/
  b. Under Browse Content, click on Platforms
  c. In the search field insert: Arabidopsis thaliana Affymetrix
  d. Click on “Samples” twice to sort by samples in descending order

This identified [ATH1-121501] Affymetrix Arabidopsis ATH1 Genome Array as the Affymetrix chip with the most available samples.

***Note:*** Try selecting an Affymetrix microarray chip that not only has a large number of open-access samples in public repositories but also includes a significant proportion of the genes of the selected organism. For example, ATH1-121501 Genome Array chip studies ∼24,000 genes, while AG Affymetrix Arabidopsis Genome Array studies only ∼8000 genes.

2. Search public repositories for ‘*Arabidopsis thaliana*’ keyword and the platform code of the microarray chip. Each repository uses a different platform code for each chip. Furthermore, alternative platform codes of the same chip and repository must also be examined.
  a. Search ArrayExpress (Kolesnikov et al., 2015) for ‘*Arabidopsis thaliana*’ and ‘A-AFFY-2’ (the platform code of ATH1-121501 in ArrayExpress) keywords.
  b. Search Gene Expression Omnibus (GEO) for ‘*Arabidopsis thaliana*’ and ‘GPL198’ (the platform code of ATH1-121501 in GEO) keywords.
  c. Download all experiments from Nottingham Arabidopsis Stock Centre (NASC) (Craigon et al., 2004) repository using the following link: https://uniofnottm-my.sharepoint.com/:f:/g/personal/sean_may_nottingham_ac_uk/Ep5b_GCihv1Nu0EYxWpkZggBK-6kAgZjMfk-9JQWJPyXUg?e=nXhIrh

**CRITICAL:** Arrange the series in different directories. Each series directory should contain the raw data (CEL files) of the samples of the series. <Series Main Directory> is the path of the directory that contains the directories of all series.

**Pause point:** Depending on the total number, study download might take a considerable amount of time.

3. Data Integrity Check.
  a. Unzip any zipped and/or gzipped CEL files, delete folders of studies which do not contain any CEL files and convert binary CEL files to text files using the following command in Ubuntu bash:
    php Parsers/Uncompress_and_Convert.php <Series Main Directory>
  b. Check if platform is “ATH1-121501” in each CEL file and delete those CEL files which are of different platforms, using:
    php Parsers/Find_Non_ATH1.php <Series Main Directory>
  c. Check and auto-delete duplicate CEL files, using:
    php Parsers/Find_CEL_duplicates.php <Series Main Directory>
    Checkpoint: When our analysis was performed (13^th^ June 2018), 19887 unique CEL files belonging to 1391 studies, were stored.

### Sample normalization

**Timing: 1d to 2 months (Varies depending on the sample number)**

The downloaded samples are normalized with Single Channel Array Normalization (SCAN). As the default Affymetrix CDF is outdated, the latest version of Brainarray (Dai et al., 2005) CDF is used. This guarantees that each probe set corresponds to a single gene and *vice versa*.

4. Download the latest version of Brainarray CDF.
  a. Visit Brainarray Custom CDF download page: http://brainarray.mbni.med.umich.edu/Brainarray/Database/CustomCDF/CDF_download.asp
  b. Visit the latest ENSG-based CDF page and copy the URL of the appropriate CDF package
    (“P” file from the “R Source Package” column, which contains the probe sequence and probe set definition data to be used by SCAN algorithm) in *Arabidopsis thaliana* ATH1121501 row.
  c. Download the CDF in RStudio, using:

install.packages("<copied url>", repos=NULL, type="source")

5. Execute SCAN on the samples with the newly downloaded CDF in RStudio, using:
  library(SCAN.UPC)
  #Custom CDF from Brainarray
  library(ath1121501atensgprobe)
  #Get each series directory
  dirseries<-list.dirs("<Series Main Directory>", full.names = TRUE)
  #Run SCAN on each series directory
  for(dirhave in dirseries [2:length(dirseries)]){
   #Set working directory
   setwd(dirhave)
   #Run SCAN
   normalised=SCAN("∗.CEL", probeSummaryPackage = ath1121501atensgprobe,
  outFilePath="SCAN_matrix.txt")
   #Show finished series
   print(dirhave)
  }
  ***Note:*** In our computer setup, SCAN needs about 2–3 min to run for each sample of Affymetrix ATH1-121501 chip. As such, SCAN total execution time depends on the total number of samples. A matrix containing all ∼20,000 genes of the CDF and their expression values in each sample of a series is produced as SCAN_matrix.txt inside each series directory.
  **Pause point:** Depending on the total number of samples, SCAN might be running for days, weeks or more than a month. The users should leave the computer running during this time, thus, the use of Uninterruptible Power Supply (UPS) is highly advisable.
  a. Navigate to ACT directory in Ubuntu terminal:
    cd ACT
    pwd
    Record the path of the ACT directory on your system. Replace it for <ACT path> in the
    following MySQL queries.
  b. Parse all SCAN_matrix.txt files to a single txt file (expression.txt) in a 4-column format, using:
    php Parsers/SCAN_Compile.php <Series Main Directory> >
    data/expression.txt
    Checkpoint: expression.txt is a tab-delimited file of *n*∙*m* lines and 4 columns, where *n* is the number of genes and *m* in the number of downloaded samples.
  c. Produce the .txt files for the tables of the database using:
    chmod 755 db_files.sh
    ./db_files.sh
  d. Insert the produced .txt files into local MySQL database in MySQL terminal using, the following commands in this order:
    LOAD DATA LOCAL INFILE '<ACT path>/data/sample.txt' INTO TABLE
    Sample;
    LOAD DATA LOCAL INFILE '<ACT path>/data/samples.txt' INTO TABLE
    Samples;
    LOAD DATA LOCAL INFILE '<ACT path>/data/ENSG.txt' INTO TABLE
    ENSG;
    LOAD DATA LOCAL INFILE '<ACT path>/data/probeset.txt' INTO
    TABLE ProbeSet;
    LOAD DATA LOCAL INFILE '<ACT path>/data/probesets.txt' INTO
    TABLE ProbeSets;
    LOAD DATA LOCAL INFILE '<ACT path>/data/selected_genes.txt'
    INTO TABLE Selected_Genes;
    LOAD DATA LOCAL INFILE '<ACT path>/data/expression.txt' INTO
    TABLE Expression;
    Checkpoint: selected_genes.txt is a single column file of *n* lines, where *n* is the number of genes which are described by the BrainArray CDF.

### Sample quality control

**Timing: 1–2 weeks**

A series of quality controls with different metrics needs to be performed to guarantee high-quality samples. In addition, samples which come from whole plant experiments or are from infected or mutated samples need to be deleted from their directory. However, in most of the cases there is no way to parse programmatically the metadata for each sample, as their format may vary significantly, which can lead to loss of important sample details. Thus, extensive manual curation is required.

6. Acquire quality control metrics for each series in RStudio, using:
  library(oligo)
  dirseries<-list.dirs("<Series Main Directory>", full.names = TRUE)
  for(dirhave in dirseries[2:length(dirseries)]){
   setwd(dirhave)
   celFiles <- list.celfiles(dirhave, full.names=TRUE)
   rawData <- read.celfiles(celFiles)
   fit1 <- fitProbeLevelModel(rawData)
   boxplot(fit1, main="NUSE", ylim = c(0.95, 1.22),outline = FALSE,
  col="lightblue", las=3, whisklty=0, staplelty=0, cex.axis=0.5)
   #export boxplot
   dev.copy(pdf,'oligo_NUSE.pdf')
   dev.off()
  a. In each series directory, PDF files containing the RLE and NUSE boxplot (Bolstad et al., 2005) for each sample of the study, are created.
  b. Samples whose RLE boxplot has an interquartile range (IQR) >0.4 or median >|0.2| (deviates from 0), are considered low-quality.
  c. Samples whose NUSE boxplot has a median >1.1 (deviates from 1), are considered low-quality.

7. A php script was created to automatically delete samples based on the aforementioned out-of-range values. Perform automatic quality control and low-quality sample deletion in Ubuntu terminal, using:

php Parsers/oligo_QC_R.php <Series Main Directory>

**Figure 2:**
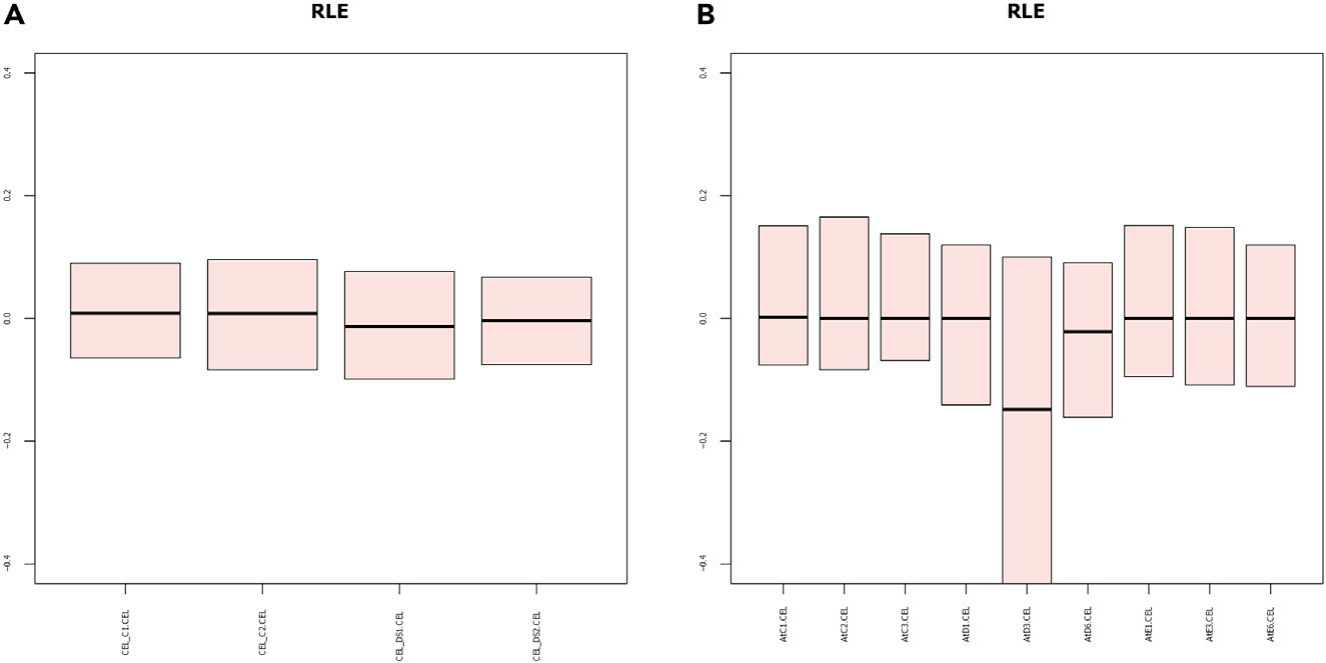
RLE plots of two ArrayExpress studies (A) All samples of E-ATMX-1 study pass the quality control. (B) AtD3.CEL sample of E-ATMX-19 study does not pass the quality control, as its IQR > 0.4, thus it must be removed from the pool of samples.

**Figure 3:**
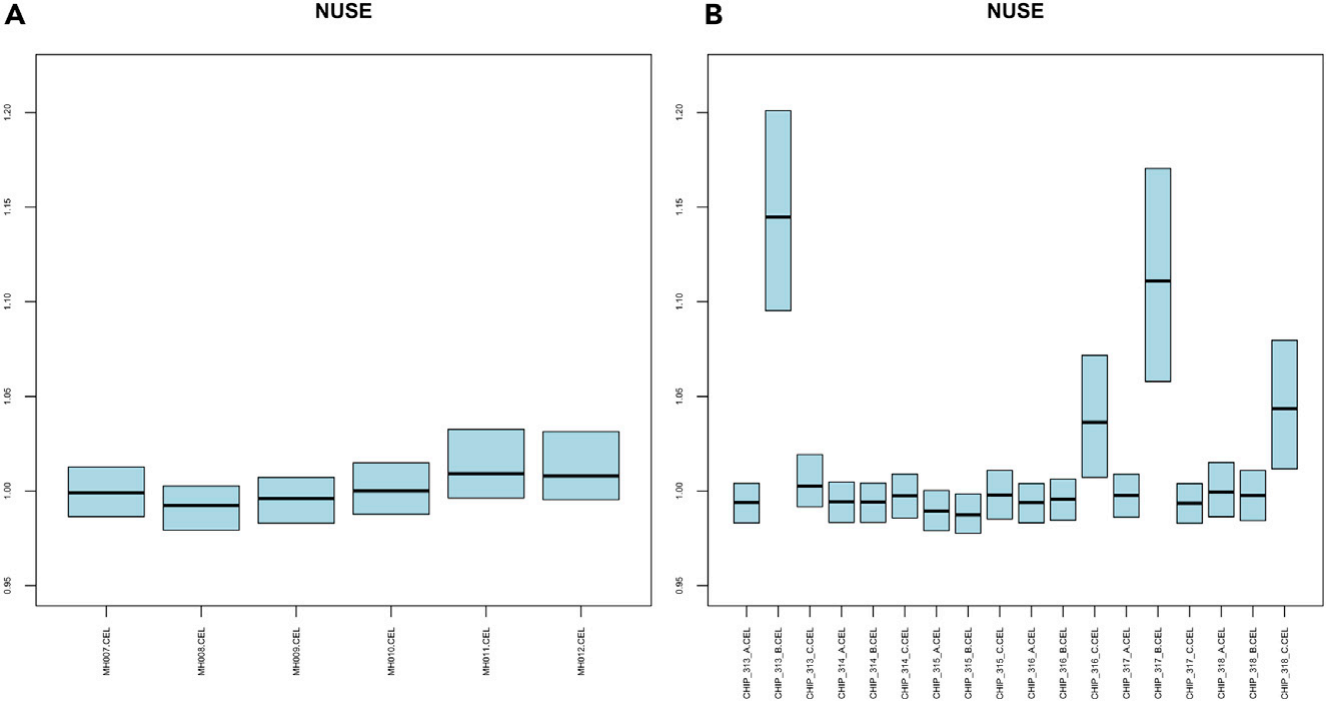
NUSE plots of two ArrayExpress studies (A) All samples of E-ATMX-7 study pass the quality control. (B) CHIP_313_B.CEL and CHIP_317_B.CEL samples E-ATMX-24 study do not pass the quality control, as their median > 1.1, thus, they should be removed from the pool of samples.

**CRITICAL:** Manual quality control is still possible, by examining the RLE (Figure 2) and NUSE (Figure 3) pdf files.

8. Download the metadata for each series (encountered as SDRF files in ArrayExpress or Series Matrix files in GEO).
  a. Manually examine the metadata looking for certain keywords and fields. Delete the CEL files of samples:
    i. of species other than Arabidopsis thaliana
    ii. that are from ‘whole plant’, ‘whole organism’, etc
    iii. of cell cultures (Table 1)
    iv. that are mutated (Table 2) or infected (Table 3)

***Note 1:*** Metadata files are available in .txt (tab delimited) format. There are certain fields such as “Tissue”, “Organism Part” or “Disease State” which are used to determine if a sample should be kept or deleted.

***Note 2:*** Since the aim of this protocol is to study the condition independent coexpression landscape of a species, the same procedure can be used for other organisms, keeping only healthy samples of distinct tissues.

**Pause point:** The users can pause and continue the metadata examination step at any point.

9. After quality control is complete, only healthy single-tissue samples remain.

Checkpoint: A large amount of samples might be deleted during the quality control process. In our case, only 6933 samples out of the 19887 remained.

10. Produce sample names using:

php Parsers/Get_Sample_Names.php <Series Main Directory> >

data/Sample_Names.txt

11. Produce selected sample index list using:
  php Parsers/Create_Selected_Samples.php data/Sample_Names.txt>
  data/Selected_Samples.txt
  a. Insert Selected_Samples.txt into Selected_Samples table in MySQL terminal, using:
    LOAD DATA LOCAL INFILE '<ACT path>/data/Selected_Samples.txt' INTO
    TABLE Selected_Samples;

**Table 1:** Selected fields of the metadata of the samples of series E-ATMX-31

Source name	Characteristics [organism]	Characteristics [OrganismPart]
Shoot 3	Arabidopsis thaliana	Shoot
Shoot 2	Arabidopsis thaliana	Shoot
Shoot 1	Arabidopsis thaliana	shoot
Cell culture 3	Arabidopsis thaliana	cultured callus
Root 3	Arabidopsis thaliana	root
Cell culture 2	Arabidopsis thaliana	cultured callus
Root 2	Arabidopsis thaliana	root
Cell culture 1	Arabidopsis thaliana	cultured callus
Root 1	Arabidopsis thaliana	root

“Cell culture 1”, “Cell culture 2” and “Cell culture 3” samples should be deleted, as they are from cell cultures, while the rest of the samples should be accepted as they are based on single tissues (“shoot” or “root”).

**Table 2:** Selected fields of the metadata of the samples of series E-ATMX-20

Source name	Characteristics [organism]	Characteristics [organism part]	Characteristics [genotype]
Zat10-OE-2	Arabidopsis thaliana	Leaf	35S:ZAT10
wildtype-2	Arabidopsis thaliana	leaf	wild type
wildtype-1	Arabidopsis thaliana	leaf	wild type
wildtype-3	Arabidopsis thaliana	leaf	wild type
Zat10-OE-1	Arabidopsis thaliana	leaf	35S:ZAT10
Zat10-OE-3	Arabidopsis thaliana	leaf	35S:ZAT10

“Zat10-OE-1”, “Zat10-OE-2” and “Zat10-OE-3” samples should be deleted, as they come from mutated plants, while “wildtype-1”, “wildtype-2” and “wildtype-3” samples should be accepted, as they are based on wild-type plants.

**Table 3:** Selected fields of the metadata of the samples of series E-GEOD-50526

Source name	Characteristics [organism]	Characteristics [organism part]	Assay name	FactorValue [genotype]	FactorValue [infect]
GSM1220728 1	Arabidopsis thaliana	leaf	GSM1220728	dde2-2	Alternaria brassicicola
GSM1220719 1	Arabidopsis thaliana	leaf	GSM1220719	dde2-2	Alternaria brassicicola
GSM1220709 1	Arabidopsis thaliana	leaf	GSM1220709	dde2-2	Alternaria brassicicola
GSM1220724 1	Arabidopsis thaliana	leaf	GSM1220724	dde2-2	Alternaria brassicicola
GSM1220714 1	Arabidopsis thaliana	leaf	GSM1220714	dde2-2	Alternaria brassicicola
GSM1220704 1	Arabidopsis thaliana	leaf	GSM1220704	dde2-2	Alternaria brassicicola
GSM1220729 1	Arabidopsis thaliana	leaf	GSM1220729	ein2-1	Alternaria brassicicola
GSM1220720 1	Arabidopsis thaliana	leaf	GSM1220720	ein2-1	Alternaria brassicicola
GSM1220710 1	Arabidopsis thaliana	leaf	GSM1220710	ein2-1	Alternaria brassicicola
GSM1220725 1	Arabidopsis thaliana	leaf	GSM1220725	ein2-1	Alternaria brassicicola
GSM1220715 1	Arabidopsis thaliana	leaf	GSM1220715	ein2-1	Alternaria brassicicola
GSM1220705 1	Arabidopsis thaliana	leaf	GSM1220705	ein2-1	Alternaria brassicicola
GSM1220730 1	Arabidopsis thaliana	leaf	GSM1220730	sid2-2	Alternaria brassicicola
GSM1220721 1	Arabidopsis thaliana	leaf	GSM1220721	sid2-2	Alternaria brassicicola
GSM1220711 1	Arabidopsis thaliana	leaf	GSM1220711	sid2-2	Alternaria brassicicola
GSM1220726 1	Arabidopsis thaliana	leaf	GSM1220726	sid2-2	Alternaria brassicicola
GSM1220716 1	Arabidopsis thaliana	leaf	GSM1220716	sid2-2	Alternaria brassicicola
GSM1220706 1	Arabidopsis thaliana	leaf	GSM1220706	sid2-2	Alternaria brassicicola
GSM1220718 1	Arabidopsis thaliana	leaf	GSM1220718	wild type	Alternaria brassicicola
GSM1220708 1	Arabidopsis thaliana	leaf	GSM1220708	wild type	Alternaria brassicicola
GSM1220727 1	Arabidopsis thaliana	leaf	GSM1220727	wild type	mock
GSM1220717 1	Arabidopsis thaliana	leaf	GSM1220717	wild type	mock
GSM1220707 1	Arabidopsis thaliana	leaf	GSM1220707	wild type	mock
GSM1220723 1	Arabidopsis thaliana	leaf	GSM1220723	wild type	Alternaria brassicicola
GSM1220713 1	Arabidopsis thaliana	leaf	GSM1220713	wild type	Alternaria brassicicola
GSM1220703 1	Arabidopsis thaliana	leaf	GSM1220703	wild type	Alternaria brassicicola
GSM1220722 1	Arabidopsis thaliana	leaf	GSM1220722	wild type	mock
GSM1220712 1	Arabidopsis thaliana	leaf	GSM1220712	wild type	mock
GSM1220702 1	Arabidopsis thaliana	leaf	GSM1220702	wild type	mock

All samples except for “GSM1220702”, “GSM1220707”, ”GSM1220712”, “GSM1220717”, “GSM1220722”, “GSM1220727” should be deleted, as they come from infected plants.

### Identification of most representative samples

**Timing: 1 day**

Selection of the most representative samples constitutes the best way to study condition-independent gene coexpression. Pearson Correlation Coefficients (PCCs, *r*-values) (Pearson, 1895) between all sample pairs are computed, using their gene expression values. Using PCC-based pairwise distances, hierarchical clustering is performed with UPGMA (Sokal and Michener, 1958) and a sample correlation tree is produced which is then automatically trimmed to contain the most representative leaf-samples.

12. PCCs between all pairs of selected samples are calculated with the following formula:

(Equation 1) $$r_{xy}=\frac{\sum_{i=1}^{n}\left(x_{i}-\bar{x}\right)\left(y_{i}-\bar{y}\right)}{\sqrt{\sum_{i=1}^{n}{\left(x_{i}-\bar{x}\right)}^{2}∙\sum_{i=1}^{n}{\left(y_{i}-\bar{y}\right)}^{2}}}$$

$x_{i}$: the expression value of gene i in sample x

$y_{i}$: the expression value of gene i in sample y

$\bar{x}$: the median expression of all genes in sample x

$\bar{y}$: the median expression of all genes in sample y

13. Calculate PCCs between sample pairs:
  a. Produce the Sample expression file with the current Selected Samples and Selected Genes in Ubuntu terminal, using:
    php Parsers/Create_Expression_R.php Samples >
    data/sample_expression.txt
    Checkpoint: sample_expression.txt is a tab-delimited file of *n*+1 lines and *m*+1 columns, where *n* is the number of genes and *m* in the number of samples that have remained after quality control.
  b. Calculate sample pairwise *r*-values and convert them to a distance value with *d = 1 – r* formula (Kassambara, 2017) in RStudio, using:
    expr_sample <- (read.table("<ACT
    path>/data/sample_expression.txt", sep = "\t", header = TRUE,
    check.names=FALSE))
    fastcor_sample <- 1 - cor(expr_sample)

14. Create the sample correlation tree with UPGMA hierarchical clustering algorithm, using the following commands in RStudio:

library("phangorn")

upgma_tree <- upgma(fastcor_sample)

write.tree(upgma_tree, "<ACT path>/data/samples_upgma.new")

15. Sort the produced sample correlation tree using Newick Utilities (Junier and Zdobnov, 2010) in Ubuntu terminal:

nw_order data/samples_upgma.new > data/samples_upgma_sorted.new

16. Prune the tree to a desired number of representative leaves (in our case the <leaf number to remain> is 3500 samples), using an in-house iterative phylogenetic algorithm pruning adjacent leaves:

php TreePrune/upgma_prune.php data/samples_upgma_sorted.new <leaf number to

remain> > data/Representative_samples.new

Those samples constitute the representative samples for the coexpression analysis.

Checkpoint: Representative_samples.new is a Newick formatted file that contains as many leaves as the number of remaining samples (in our case 3500).

17. Obtain the leaf-sample names using:

php TreePrune/Tree_Names.php data/Representative_samples.new >

data/Representative_Sample_Names.txt

and produce Selected Samples indexes using:

php Parsers/Create_Selected_Samples_from_Leaf_names.php

data/Representative_Sample_Names.txt >

data/Representative_Selected_Samples.txt

18. Empty Selected_Samples table in MySQL terminal, using:

truncate table Selected_Samples;

then insert Representative_Selected_Samples.txt into Selected_Samples table of local MySQL database using:

LOAD DATA LOCAL INFILE '<ACT

path>/data/Representative_Selected_Samples.txt' INTO TABLE

Selected_Samples;

**Pause point:** The users can pause before performing the final step of the protocol.

### Gene coexpression tree creation

**Timing: 2 days**

By calculating the pairwise Pearson Correlation coefficients between all gene pairs from the representative samples, we can create a gene coexpression distance matrix which will be used as input for the construction of the gene coexpression tree.

19. PCC-based distances between all pairs of selected genes are calculated with the previously mentioned formula (Equation 1). However, in this case:
  $x_{i}$: the expression value of gene x in sample i
  $y_{i}$: the expression value of gene y in sample i
  $\bar{x}$: the median expression of gene x in all samples
  $\bar{y\;}$: the median expression of gene y in all samples

20. Calculate PCCs between gene pairs:
  a. Produce the Gene expression file with the current Selected Samples and Selected Genes in Ubuntu terminal, using:
    php Parsers/Create_Expression_R.php Genes > data/gene_expression.txt
    Checkpoint: gene_expression.txt is a tab-delimited file of *m*+1 lines and *n*+1 columns, where *n* is the number of genes and *m* in the number of representative samples.
  b. Calculate gene pair *r*-values and convert them to a distance value in RStudio, using:
    expr_gene <- (read.table("<ACT path>/data/gene_expression.txt",
    sep = "\t", header = TRUE, check.names=FALSE))
    fastcor_gene <- 1 - cor(expr_gene)

21. Create the gene coexpression tree with UPGMA hierarchical clustering algorithm, using the following commands in the same session of RStudio:

library("phangorn")

upgma_tree <- upgma(fastcor_gene)

write.tree(upgma_tree, "<ACT path>/data/genes_upgma.new")

22. Sort the tree using Newick Utilities in Ubuntu terminal:

nw_order data/genes_upgma.new > data/genes_upgma_sorted.new

Checkpoint: genes_upgma_sorted.new is a Newick-formatted file that contains as many leaves as the number of genes.

23. Visualize the tree in Dendroscope:
  a. Open <ACT path>/data/genes_upgma_sorted.new in Dendroscope
  b. Select Layout > Draw tree or network as rectangular phylogram
  c. Untick View > Sparse Labels
  d. Press Ctrl+F or click on the Binoculars icon in the Dendroscope toolbar to search for an AGI code of a gene of interest
  e. Paste the AGI code on the search field and press Enter. The leaf that corresponds to the gene of interest will be highlighted in yellow
  f. Using the mouse wheel, zoom to a subtree node containing the highlighted gene of interest
  g. Left-click the last common ancestral node
  h. Select Select > Advanced Selection > Select Subnetwork
  i. Select Select > Invert Selection
  j. Select Edit > Delete Taxa
  k. Export the subtree as Newick by clicking File > Export > Newick and saving it as a .new file
  l. The gene list can be extracted from the subtree file using:

php <ACT>TreePrune/Tree_Names.php <subtree newick file> >

subtree_gene_list.txt

Checkpoint: subtree_gene_list.txt is a single column file of *s* lines, where *s* is the number of leaves of the subtree Newick-formatted file exported by Dendroscope.

## Expected outcomes

**Figure 4:**
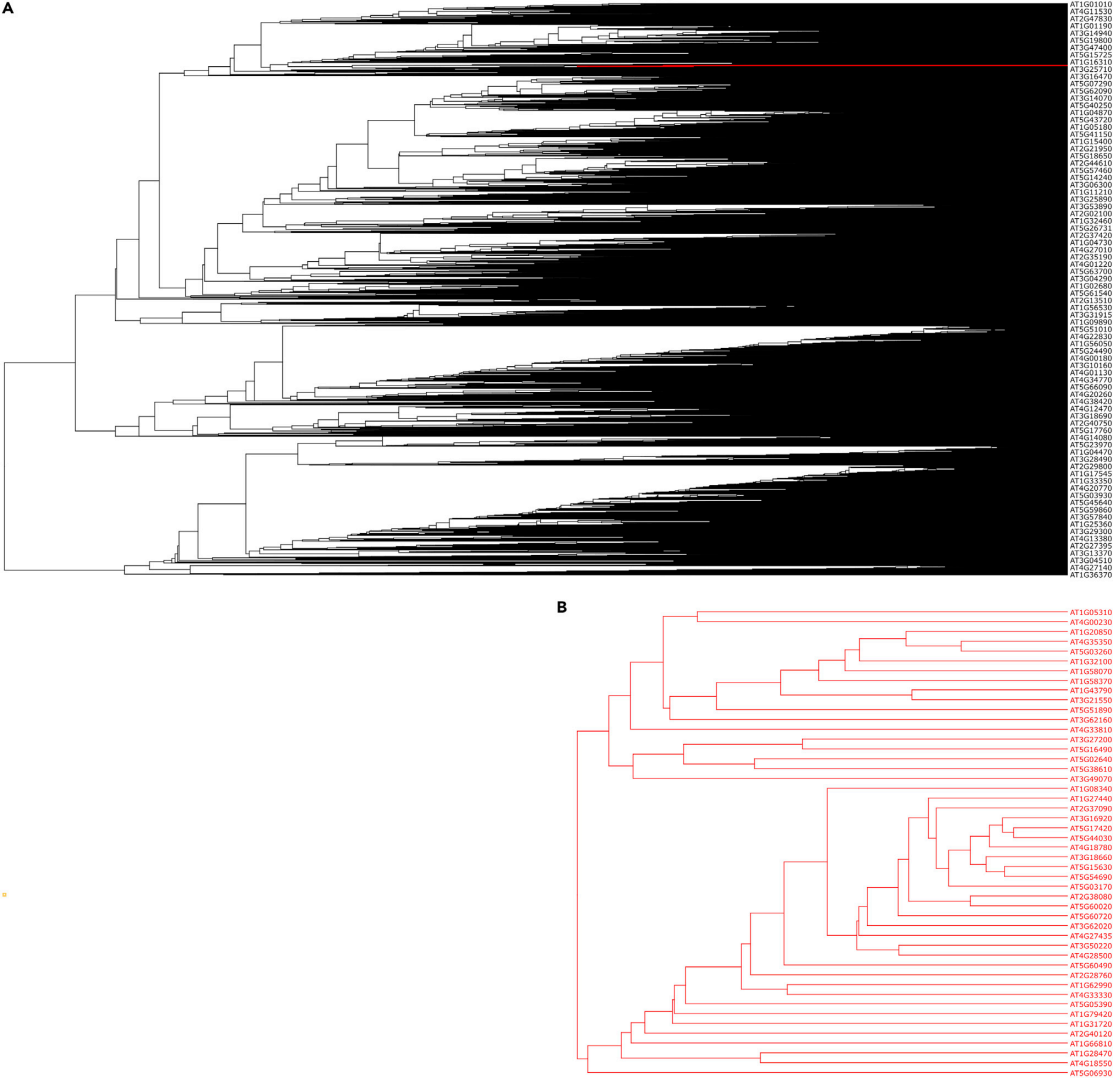
*Arabidopsis thaliana* gene coexpression trees as viewed by Dendroscope (A and B) Node labels are the AGI codes of the *Arabidopsis thaliana* genes. (A) Coexpression tree containing all *Arabidopsis thaliana* genes. The leaves highlighted in red denote the region where CTL2 (AT3G16920) coexpression subtree is located (B) Gene coexpression subtree containing CTL2 and its coexpressed genes.

The outcome of the protocol is a gene coexpression phylogenetic tree (Figure 4A) as a Newick-formatted file. In this example, the output tree contains 20,430 *Arabidopsis thaliana* genes which are represented as leaves. Coexpressed genes that may constitute functional partners and, thus, share similar biological functions and metabolic pathways, are grouped together in the same subclade. The tree itself can be viewed by various phylogenetic software supporting Newick-formatted trees.

The list of the genes of a subtree can be used as input in downstream analyses, such as functional network analysis or enrichment analysis. By examining the enriched gene terms of that subtree, new biological functions for those gene sets may be discovered. In addition, it is possible to attribute biological roles to neighboring genes of unknown function.

Functional partners of a gene of interest may be found in its neighboring leaves. In the example coexpression subtree (Figure 4B), *CTL2* (shown by its AGI code: AT3G16920), is grouped with genes which are associated with plant-type secondary wall biogenesis.

## Quantification and statistical analysis

Enrichment analysis can discover the predominant biological processes of a given coexpressed gene list:

1. String (Szklarczyk et al., 2021) creates a protein-protein interaction (PPI) network using a gene list as input:
  a. Visit the String website (https://string-db.org/) and click on “Search”
  b. Click on “Multiple Proteins”
  c. Paste the contents of subtree_gene_list.txt file that was created previously to “List Of Names” field and select the Organism (in this case *Arabidopsis thaliana*)
  d. Click on “Search”
  e. Click on “Continue”
  f. The resulting PPI network of the input genes is displayed
  g. In “Analysis” tab, “Network Stats” show the density of the network and consequently indicate the degree of functional interaction between the input genes. “Functional enrichments in your network” show the predominant biological terms which are associated with the coexpressed genes.
  h. In “Settings” tab, network parameters, such as interaction sources and interaction thresholds, can be adjusted.
2. WebGestalt (Liao et al., 2019) performs enrichment analyses for a gene list:
  a. Visit the WebGestalt website (http://www.webgestalt.org/)
  b. In Basic Parameters:
    i. Select “Organism of Interest” (in this case *Arabidopsis thaliana*)
    ii. Select “Over-representation analysis (ORA)” as “Method of Interest”
  c. Select all available or a certain combination of “Functional Databases” by clicking on the plus mark symbol on the left of the field.
  d. In Gene List:
    i. Select “Gene Symbol” as GeneID type
    ii. Paste the contents of the subtree_gene_list.txt file that was created previously.
  e. In Reference Gene List:
    i. Select “Gene Symbol“ as GeneID type
    ii. Select “Upload Gene List” and use ACT/data/selected_genes.txt file as input
  f. Click on “Submit”
    i. Click on “Table” tab for table view. The enriched biological terms of the coexpressed genes to the gene interest included in the subtree, are shown.

## Limitations

The main limitation of this protocol originates from the transcriptomic technology of microarrays which is not able to study the expression of genes for which no probe is available. Furthermore, cross-hybridization may make false estimations of the gene expression, distorting correlation between members of the same family of genes and other genes. Thus, RNA-seq, having greatly advanced in the latest years, has replaced microarrays as transcriptomic technology of choice, to a large extent. RNA-seq has higher sensitivity and there is a growing amount of data available in public repositories. Nevertheless, it is shown that microarray and RNA-seq-based coexpression analyses produce comparable gene coexpression networks (Malatras et al., 2020; Obayashi et al., 2018). Considering the fact that there is no definitive pipeline to perform coexpression analysis from raw RNA-seq data, microarray data are still relevant, as their normalization algorithms have been tested and perfected throughout the technology’s lifetime.

A limitation of gene coexpression tree depiction, used in this protocol, is the fact that it cannot portray anti-coexpressed genes. As gene pairwise *r*-values are transformed to non-negative distance values, anti-correlated genes are not inferred. Finally, this depiction assumes that one gene may only participate in a single group of functional partners. This limitation contradicts the already known fact that genes may interact with different gene subgroups which are related to different functions.

As far as the execution of this protocol is concerned, advanced programming and database management knowledge is required.

## Troubleshooting

### Problem 1

RStudio crashes/displays errors during the creation of the coexpression tree of genes (step 21).

### Potential solution

First, make sure that the matrix is formatted correctly for Phangorn and that there are no missing values in the matrix.

When trying to calculate the correlations or produce a tree using a large number of genes (>20,000), R requires a lot of RAM (possibly more than the recommended 64 GB). We recommend closing all other applications that might use memory resources. If the problem persists, the only solution would be to increase the available RAM of the machine.

### Problem 2

Error during the installation of Bioconductor packages.

### Potential solution

This protocol assumes that the latest available R version is used. However, at some point in time, certain packages may stop being supported. In such case, we recommend installing a (older) version of R that supports the installation of those packages.

### Problem 3

Oligo package does not support the creation of additional quality control metrics (apart from RLE and NUSE boxplots) for downloaded samples (step 6).

### Potential solution

We propose installing the following packages to produce NUSE and RLE boxplots and Quality Control reports (saved as AffyQCReport.pdf). However, those are only available for non-exon arrays microarray platforms and for older versions of R (<4.0.3).

simpleaffy (Miller, 2018):

if (!requireNamespace("BiocManager", quietly = TRUE))

 install.packages("BiocManager")

BiocManager::install("simpleaffy")

affyPLM (Bolstad et al., 2005; Brettschneider et al., 2008):

if (!requireNamespace("BiocManager", quietly = TRUE))

 install.packages("BiocManager")

BiocManager::install("affyPLM")

affyQCReport (Parman et al., 2021):

if (!requireNamespace("BiocManager", quietly = TRUE))

 install.packages("BiocManager")

BiocManager::install("affyQCReport")

Quality control is performed using the following commands:

library(affyQCReport)

library(simpleaffy)

library(affyPLM)

#Get each series directory

dirseries<-list.dirs("<Series Main Directory>", full.names = TRUE)

for(dirhave in dirseries[2:length(dirseries)]){

 setwd(dirhave)

 #read all CEL files from current working directory

 readdata <- ReadAffy(compress = FALSE)

 #first Quality Assessment

 Saqc <- QCReport(readdata)

 dataPLM <- fitPLM(readdata,output.param=list(varcov="none"))

 par(mar = c(9, 4, 4, 2) + 0.1)

 boxplot(dataPLM, main="NUSE", ylim = c(0.95, 1.22),

 outline = FALSE, col="lightblue", las=3,

 whisklty=0, staplelty=0, cex.axis=0.5)

 #export boxplot

 dev.copy(pdf,'NUSE.pdf')

 dev.off()

 Mbox(dataPLM, main="RLE", ylim = c(-0.4, 0.4),

 outline = FALSE, col="mistyrose", las=3,

 whisklty=0, staplelty=0, cex.axis=0.5)

 #export Mbox

 dev.copy(pdf,'RLE.pdf')

 dev.off()

 print(dirhave)

}

### Problem 4

Newick Utilities cannot be installed/run on my system.

### Potential solution

In this protocol, we suggest downloading an already compiled version of Newick Utilities, which is, however, available only through the Wayback Machine. If the link becomes dead, or any other problem occurs, we recommend visiting the official GitHub page of the software (https://github.com/tjunier/newick_utils). Alternatively, other software, such as Dendroscope, can be used for tree sorting.

### Problem 5

The coexpression tree cannot load in Dendroscope (step 23).

### Potential solution

Phylogenetic trees with more than 30,000 leaves, require larger amounts of RAM to open in Dendroscope. We suggest increasing the available RAM of Dendroscope or using another tree visualization software.

### Problem 6

There is an issue with one or more PHP scripts that are included in the ACT GitHub folder.

### Potential solution

The user can report the issue through GitHub.

## Resource availability

### Lead contact

Further information and requests for resources should be directed to and will be fulfilled by the lead contact, Ioannis Michalopoulos (imichalop@bioacademy.gr).

### Materials availability

This study did not generate new unique reagents.

## Data Availability

The microarray samples analyzed during the current study are available at: https://data.mendeley.com/datasets/hgvk669v89/ Custom scripts and data used are available in: https://github.com/imichalop/ACT Any additional information required to reanalyze the data reported in this paper is available from the lead contact upon request.
